# Targeting TIGIT for cancer immunotherapy: recent advances and future directions

**DOI:** 10.1186/s40364-023-00543-z

**Published:** 2024-01-16

**Authors:** Peng Zhang, Xinyuan Liu, Zhuoyu Gu, Zhongxing Jiang, Song Zhao, Yongping Song, Jifeng Yu

**Affiliations:** 1 Department of Thoracic Surgery, the First Affiliated Hospital of Zhengzhou University, Zhengzhou, 450052 Henan China; 2 Henan Medical Key Laboratory of Thoracic Oncology, Zhengzhou, 450052 Henan China; 3 Institute of Biomedical Informatics, Bioinformatics Center, Henan Provincial Engineering Center for Tumor Molecular Medicine, School of Basic Medical Sciences, Henan University, Kaifeng, 475004 Henan China; 4 Department of Hematology, the First Affiliated Hospital of Zhengzhou University, Zhengzhou, 450052 Henan China; 5 Henan International Joint Laboratory of Nuclear Protein Gene Regulation, Henan University College of Medicine, Kaifeng, 475004 Henan China

**Keywords:** TIGIT, Target immunotherapy, Immune checkpoint pathway, cancer immunotherapy, Clinical trial

## Abstract

As a newly identified checkpoint, T cell immunoreceptor with immunoglobulin and tyrosine-based inhibitory motif (ITIM) domain (TIGIT) is highly expressed on CD4^+^ T cells, CD8^+^ T cells, natural killer (NK) cells, regulatory T cells (Tregs), and tumor-infiltrating lymphocytes (TILs). TIGIT has been associated with NK cell exhaustion in vivo and in individuals with various cancers. It not only modulates NK cell survival but also mediates T cell exhaustion. As the primary ligand of TIGIT in humans, CD155 may be the main target for immunotherapy due to its interaction with TIGIT. It has been found that the anti-programmed cell death protein 1 (PD-1) treatment response in cancer immunotherapy is correlated with CD155 but not TIGIT. Anti-TIGIT alone and in combination with anti-PD-1 agents have been tested for cancer immunotherapy. Although two clinical studies on advanced lung cancer had positive results, the TIGIT-targeted antibody, tiragolumab, recently failed in two new trials. In this review, we highlight the current developments on TIGIT for cancer immunotherapy and discuss the characteristics and functions of TIGIT.

## Introduction

Immune checkpoint inhibitors (ICIs) function by restoring the induction, activation, and expansion of tumor-specific cytotoxic T cells, resulting in durable therapy responses [1–4]. The Food and Drug Administration (FDA) has approved a number of ICIs targeting programmed cell death protein 1 (PD-1), cytotoxic T lymphocyte antigen-4 (CTLA-4), and lymphocyte activation gene-3 (LAG-3) for the treatment of various cancers, such as non-small cell lung cancer (NSCLC), melanoma, carcinoma of the head and neck, and hematological malignancies [1, 5–9]. There are still a large number of patients who do not respond well to this treatment, and the reason may be low expression of programmed death-ligand 1 (PD-L1), a “cold” tumor microenvironment (TME) characterized by a paucity of effector T cells or a surplus of immune regulatory cells [9–12]. Furthermore, innate and acquired resistance to such treatment remains a major obstacle [13].

Following the emergence of these well-known ICIs, a novel checkpoint, T cell immunoreceptor with immunoglobulin and tyrosine-based inhibitory motif (ITIM) domain (TIGIT), also known as WUCAM, VSTM3, or VSIG9, has been identified [14–17]. Clinical trials of anti-TIGIT or anti-TIGIT and anti-PD-1 combinations are being done. Despite being successful in a few clinical trials [18–22], the TIGIT-targeted antibody tiragolumab recently failed to reach the endpoint in patients with advanced lung cancers [23, 24]. In this review, we discuss the characteristics and functions of TIGIT activity. We looked further into the potential reasons why anti-TIGIT experiments in both preclinical and clinical settings failed.

## TIGIT: expression, function and the signaling pathway

### The expression and function of TIGIT

TIGIT is extensively overexpressed on tumor-infiltrating lymphocytes (TILs) such as CD8^+^ T cells, CD4^+^ T cells, regulatory T cells (Tregs), and natural killer (NK) cells across various malignancies [25–31]. Interestingly, TIGIT was found to be preferentially expressed on the CD16^+^ NK cell subpopulation in contrast to other PVR-like receptors that are expressed on human NK cells [32]. Research indicated that intratumoral NK and T cells expressed more TIGIT than peripheral blood NK and T cells did [33, 34], TIGIT^+^ CD4^+^ T cells are enriched in chronic lymphocytic leukemia (CLL) but not CD8^+^ T cells [35]. These suggest that the role of TIGIT may vary between solid tumors and hematological cancers. Additionally, in a number of malignancies, the expression of TIGIT was associated with tumor stage, survival, and the TILs component [33, 36–38].

Advancements in chimeric antigen receptor T-cell (CAR-T) therapy aim to enhance chemotaxis and infiltration into the TME, while efforts continue to reduce cell exhaustion and treatment-related toxicities [39]. Several CAR-T cell products have been approved for treating hematological malignancies [40–43]. However, some patients remained poor responders or relapsed with CAR-T therapy [44]. A recent study showed an improvement in CAR-T efficacy with TIGIT inhibition alone based on the sequential analysis of manufactured and infused CAR-T using single-cell RNA and protein expression data [45]. Targeting TIGIT may prevent CAR-T-related relapses and thus promote long-term progression-free survival in mantle cell lymphoma (MCL) patients, according to another study that found that over-expression of TIGIT played a critical role in T-cell suppression associated with CAR-T relapse in mantle cell lymphoma patients [44].

In a typical scenario, the co-expression of co-inhibitory receptors serves as an indicator of immune cell exhaustion, a state characterized by a diminished response to antigen stimulation. For instance, elevated co-expression of TIGIT and T cell immunoglobulin and mucin-domain-containing-3 (TIM-3), on NK cells was found in patients with hepatitis B virus-associated hepatocellular carcinoma (HBV-HCC) [46]. This specific subset of NK cells displayed compromised functionality, as evidenced by a reduction in cytotoxicity, cytokine release, and cell proliferation - all hallmarks of an “exhausted” phenotype [46]. Importantly, these TIGIT^+^ TIM-3^+^ NK cells have been implicated in the progression of HBV-HCC, underscoring their significant role in the pathogenesis of this disease [46]. Tumor-infiltrating NK cells (TiNKs) are more dysfunctional compared to circulating human NK cells. Interestingly, while TIGIT^+^ NK cells demonstrated a higher lytic potential, they paradoxically exhibit lower actual lytic activity against CD155^+^ major histocompatibility complex (MHC)-class I deficient melanoma cells than their TIGIT- counterparts [34]. An increased percentage of NK lacking CD226 expression and co-expression of TIGIT and CD96 are associated with better survival in AML patients [47].

TIGIT not only influences the survival and exhaustion of NK cells, as observed in animal models and cancer patients, but it also actively mediates T cell depletion. TIGIT functions as a coinhibitory receptor and is typically co-expressed with PD-1 in CD8^+^ and CD4^+^ T effector memory cells, where it is associated with T cell exhaustion [36, 48–53]. Additionally, TIGIT and PD-1 expression displayed diminished effector functionality of intratumoral T-cells in B-cell non-Hodgkin lymphoma (NHL) and predicted survival in lung squamous cell carcinoma (LUSC) [36, 48]. Therefore, TIGIT and PD-1 may serve as potential predictive indicators for dual-targeting immunotherapy. According to reports, both the quantity of Tregs and the expression of TIGIT were associated with the TILs hypofunction [54]. In addition, TIGIT expression represents a sign of T cell exhaustion in several cancers [55–57]. Elevated CD8^+^ TIGIT^+^ T cells exhibiting signs of exhaustion have been associated with less favorable clinical outcomes in HBV-HCC [27]. Meanwhile, intratumoral TIGIT^+^ CD8^+^ T-cell abundance could serve as an independent prognosticator for clinical outcome and a predictive biomarker [58], and TIGIT and PD-1 expression atlas predicts response to adjuvant chemotherapy and PD-L1 blockade in muscle-invasive bladder cancer [59]. Furthermore, TIGIT-expressing CD4^+^ T cells represent a tumor-supportive T cell subset in CLL [35] and TIGIT blockade can restore CD8^+^ T-cell immunity against multiple myeloma (MM) [60]. Blocking TIGIT/CD155 signalling reverses CD8^+^ T cell exhaustion and enhances the antitumor activity in cervical cancer [56]. Additionally, the secretion of IL-10 from CD8^+^ T cells can in turn increase TIGIT expression, thereby further exacerbating immunosuppression [61].

Unlike other T cells, the presence of the TIGIT marker on Tregs is linked with consistent FOXP3 activity, nuclear localization of FOXO1, and enhanced suppressive functionality [62]. From a mechanistic perspective, TIGIT functions as a transcriptional target of FOXP3. And the suppression of the PI3K/AKT/mTOR pathway aids in preserving the identity and stability of Tregs, resulting in the nuclear retention of FOXO1 [62]. Additionally, TIGIT^+^ Tregs form a distinct subset, uniquely defined by their specific capacity to suppress T helper cell (Th)1 and Th17 pro-inflammatory responses, as well as to inhibit the proliferation of effector T cells [63].

**Fig. 1:**
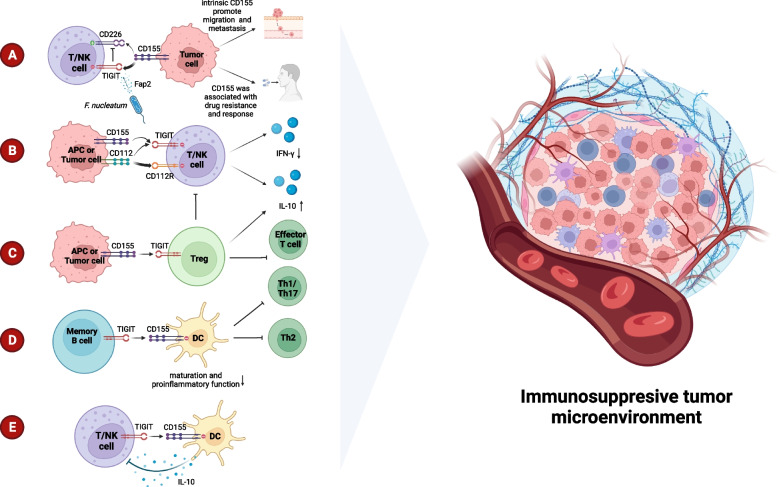
Mechanisms of immunosuppression of TIGIT in TME. A. CD155 binds with TIGIT with higher affinity compared to CD226 leading to deactivation of T or NK cells. In addition, CD155 is associated with drug resistance, and tumor cell migration and metastasis. Fap2, secreted from *F. nucleatum,* triggers inhibition by binding to TIGIT. B. The interaction of CD112 and CD112R, or TIGIT, inhibits proliferation of T-cells and NK cells by reducing IFN-γ production. CD155 increases IL-10 production by binding to TIGIT. C. TIGIT on Treg activates its immunosuppression on Th1, Th17, and effector T cells. D. The binding of TIGIT and CD155 on memory B cells exhibits deactivated DCs and inhibits Th1/17/2. E. The production of IL-10 from DCs inhibits TILs

The TIGIT^+^ CD20^+^ B cells observed infiltrating tumors and tertiary lymphoid structures (TLS) were found to be significantly correlated with survival rates as well as the response to adjuvant chemotherapy [64]. Besides, TIGIT on memory B cells regulates the immune response by directly influencing T cells and inhibiting DC proinflammatory function. This suppresses Th1, Th2, and Th17 immune responses and CXCR5^+^ICOS^+^T cell responses while promoting the immune regulatory function of T cells. TIGIT^+^ memory B cells are also superior to other B cells at expressing additional inhibitory molecules, including IL-10, TGFβ1, granzyme B, PD-L1, CD39/CD73, and TIM-1 [65]. The mechanisms of immunosuppression mentioned in TIGIT were elucidated in Fig. 1.

**Fig. 2:**
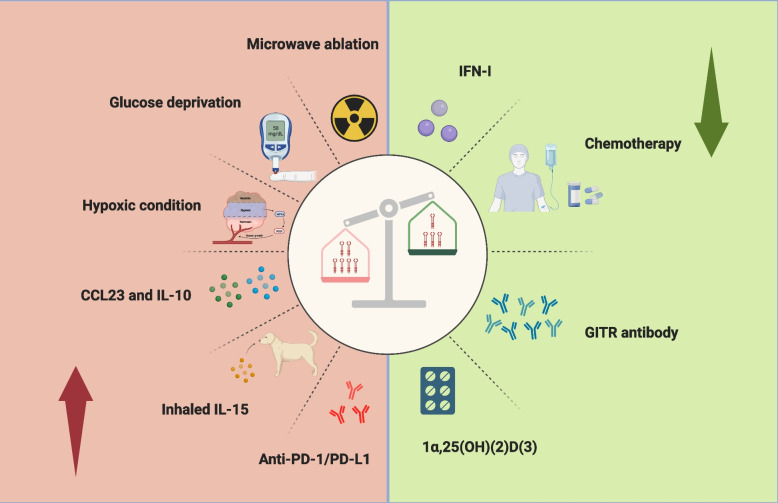
The expression of TIGIT can be influenced by a variety of factors. Notably, Interferon-I (IFN-I), chemotherapy agents, GITR antibodies, and 1α,25-Dihydroxyvitamin D3 (1α,25(OH)(2)D(3)) have been observed to downregulate its expression. In contrast, microwave ablation, glucose deprivation, hypoxic conditions, as well as the presence of CCL23, IL-10, inhaled IL-15, and anti-PD-1/PD-L1 therapies have been found to enhance the expression of TIGIT, thereby contributing to an immunosuppressive TME

Generally, the decreased TIGIT expression inhibited tumor growth in mice and activated IFN-γ secretion by NK and CD8^+^ T cells [66]. In renal and liver allograft patients, a reduction or absence of TIGIT^+^ memory B cells is linked to heightened donor-specific antibody and T follicular helper cell (TFH) responses while simultaneously being associated with diminished Treg responses [65]. A fascinating study has shed light on the inhibitory role of TIGIT within cells [67]. Making use of chimeric costimulatory switch receptors (CSR), the research successfully managed to transmute coinhibitory signals in T cells into those that advance their activity. The CSR utilized for this purpose was ingeniously constructed by fusing the extracellular domain of TIGIT with the signaling domain of CD28 [67]. Previous research has revealed that the expression of TIGIT could vary, influenced by a multitude of factors [33, 68–71] (Fig. 2). In addition to its role in infectious disorders, Sumida et al. identified that type 1 interferon (IFN-I) also inhibited the expression of TIGIT in vitro [72]. Notably, chemotherapy was found to reduce the presence of TIGIT on CD8^+^ T cells in gastric cancer cases [68, 73], while it was observed to increase following microwave ablation [71]. Factors such as glucose deprivation and hypoxic conditions were also identified as triggers for an upsurge in TIGIT expression on tumor cells [70]. Additionally, the C-C motif chemokine ligand 23 (CCL23), typically secreted by macrophages, may play a role in fueling the growth of cancer cells [69, 74]. In an insightful discovery made by Kamat, K., and his team, they noted that CCL23 contributed to the increased expression of TIGIT on CD8^+^ T cells through a process involving GSK3β phosphorylation [69]. In related findings, dogs diagnosed with metastatic osteosarcoma and treated with inhaled IL-15 showed not only an elevation in activation markers but also a noticeable rise in TIGIT levels [33].

### The structure of TIGIT

The TIGIT gene, which encodes a transmembrane protein with 244 amino acids, is found on human chromosome 3q13.31 [17]. It was first identified in 2009 by bioinformatics research that sought to identify an inhibitory receptor [14]. Together with CD96 (TACTILE) and CD226 (DNAM-1), it is a member of the immunoglobulin superfamily of receptors, and TIGIT also forms part of the poliovirus receptor (PVR)/nectin family, which includes PVR (CD155) and Nectin-2 (CD112) [75, 76]. Structurally, TIGIT is composed of three parts: an extracellular immunoglobulin variable-set (IgV) domain, a type I transmembrane domain, and a highly conserved intracellular inhibitory domain that contains an ITIM and an immunoglobulin tyrosine tail (ITT) motif, features that are consistent in both mice and humans [14, 77].

The (V/I)(S/T)Q, AX_6_G, and T(F/Y)P submotifs in the TIGIT IgV domain mediate a ‘lock and key’ *trans*-interaction with *cis*-homodimers of PVR [14, 77, 78]. These conserved submotifs were identified in the PVR/nectin family, including CD226, CD96, CD112R, CD155, CD112, and CD113 [77]. Within the intracellular inhibitory domain, ITT-like and ITIM motifs, two conserved inhibitory domains, exert slightly distinct functions. By phosphorylating either the tyrosine residues of the ITIM (Y277) or the ITT-like motif residue (Y233), in mice, the inhibitory activity of TIGIT can be triggered [16]. Whereas in humans, the ITIM motif mediates a weak inhibitory signal [79, 80]. The ITT-like motif is phosphorylated at Tyr225 and binds to cytosolic adapters Grb2 and β-arrestin 2 and is further responsible for the recruitment of the Src homology 2 (SH2) domain containing inositol polyphosphate 5-phosphatase 1 (SHIP1) [56, 81, 82] which impairs tumor necrosis factor (TNF) receptor-associated factor 6 (TRAF6) auto-ubiquitination to abolish NF-kB and ERK activation [56, 81] and terminates PI3K/MAPK signaling [79, 83], leading to suppression of NK cell and CD8^+^ T cell function.

### The ligands and pathways of TIGIT

TIGIT mainly interacts with four ligands, nectin and nectin-like adhesion molecules, namely CD155 (PVR, or Necl-5), CD112 (PVRL2, Nectin-2), CD113 (PVRL3, Nectin-3), and nectin-4 (PRR4, PVRL4) [83, 84]. In both humans and mice, CD155 acts as the principal ligand for TIGIT [28]. This is primarily due to its superior affinity when compared with CD112 and CD113 [14]. TIGIT and CD226 (DNAM-1) are competitors for the same ligands, CD155 and CD112 [85]. It was discovered that a mutation from Ala to Thr at residue 67 of CD155 increased the binding affinity for TIGIT [86]. Another CD155 receptor with a different affinity from TIGIT is CD96 (TACTILE) [78].

**Fig. 3:**
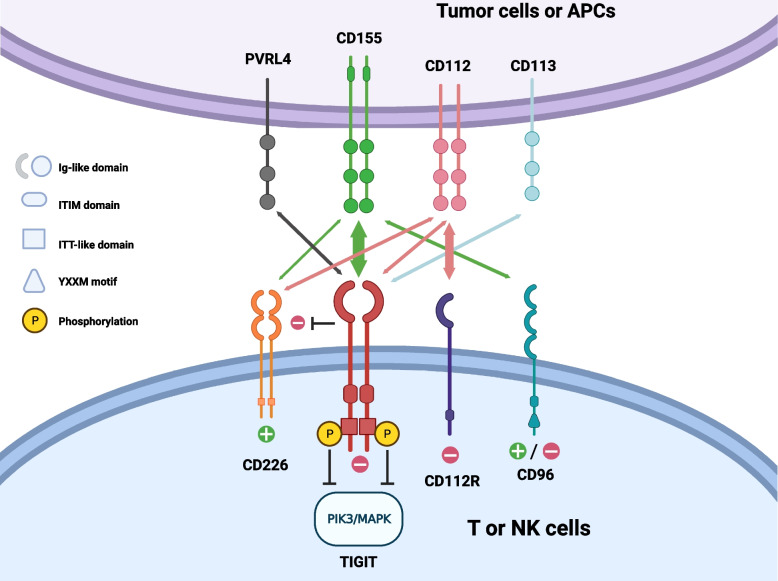
The interaction of TIGIT-related ligands and receptors. PVRL4, CD155, CD112, and CD113 are expressed on tumor cells or APCs as ligands, and CD226, TIGIT, CD96, and CD112R act as receptors on T cells or NK cells. Every receptor here, apart from CD226 which uniquely possesses an ITT-like domain, features an intracellular ITIM domain. TIGIT has an ITT-like motif, and CD96 contains a YXXM motif. TIGIT binds to CD155 with a higher affinity and inhibits the interaction of CD226 and CD155. In humans, the inhibition of TIGIT mainly relies on the phosphorylation of an ITT-like motif and ultimately terminates PI3K/MAPK signaling. However, the downstream function of CD96 has not been clear until now. The arrow’s thickness represents the affinity of ligands and receptors

CD155, as the PVR and the fifth member of the nectin-like molecule family, demonstrated different immune efficacy when combined with its ligands CD226, TIGIT and CD96 [83, 87, 88]. By interacting with phosphorylated TIGIT, which was controlled by recruiting SHIP1 and then blocking PI3K/MAPK signaling as previously described (Fig. 3) [83, 89, 90], CD155 exhibits immunosuppressive properties [83]. Conversely, when CD155 binds with CD226, it activates NK cells and T cells [83, 90]. Compared to healthy tissues, malignancies have higher levels of CD155 expression [29, 83, 89, 91, 92]. Follicular dendritic cells, tumor-associated macrophages (TAMs), and other tumor-infiltrating myeloid cells (TIMs) but not tumor-infiltrating lymphocytes (TILs) from multiple tumors have been found to express CD155 in the TME [25, 28, 50, 86, 91]. Intriguingly, a biomarker for predicting a worse prognosis and stage is CD155 high expression [38, 93]. The intrinsic functions of CD155 promote tumor cell migration and metastasis [89, 90]. Targeted TIGIT or CD155 therapy enhances immune cell activation [93]. Kawashima et al. found that in patients who demonstrated a positive response to ICIs, an increase in CD155 expression was observed in tumor cells that survived after TILs. This escalated expression of CD155 led to the suppression of T-cell activation, particularly those expressing TIGIT [25]. Currently, it is possible to use CD155 as an immunotherapy target because of its interaction with activating or inhibiting receptors [92]. In melanoma patients with an inflamed TME, CD155 was associated with ICI resistance, including both primary and acquired resistance [25]. Meanwhile, Jiang, C. et al. discovered that the response to anti-PD-1 is correlated to CD155 expression rather than TIGIT expression [94].

Research has previously indicated that CD112 predominantly resides at the adherent junctions of epithelial cells and is expressed universally across various cell types, including but not limited to follicular dendritic cells (DCs), monocytes, and multiple cancer cells [50, 95–97]. Notably, its association with clinical outcomes may vary based on the type of tumor and where it is expressed [97]. By binding to different ligands, CD112 exerts the opposite function. CD112-CD226 can promote T cell proliferation and NK cell-mediated cytotoxicity [87, 98]. On the contrary, when attached to TIGIT (despite its low affinity) and CD112R (PVRIG), CD112 inhibits proliferation of T-cells and NK cells by reducing interferon-γ (IFN-γ) production and NK cell-mediated cytotoxicity [87, 97]. Moreover, it is noteworthy that TIGIT/CD112R pathways may also be upgraded as potential immunotherapy targets [87].

CD226 is a co-stimulatory receptor that shares common ligands, specifically CD155, with TIGIT and CD96. It is expressed on T cells, NK cells, NK/T cells, B cells, monocytes/macrophages, DCs, megakaryocytes/platelet lineage hematopoietic precursor cells, endothelial cells, and mast cells [75, 99–102]. In the context of cancer, the interaction between CD226 and CD155 is crucial for activating anti-tumor immune responses. When CD226 expression was low or absent, CD8^+^ TIL exhibited poor activation and malfunction, respectively [103, 104]. Interestingly, compared to the NK cells in the peripheral blood of healthy donors, those derived from ascites in patients with advanced ovarian cancer (OC) exhibited a decreased expression of the activating receptor CD226 [105], which implies that the anti-tumor functionality of CD226 might rely on both extra- and intra-tumoral heterogeneity, akin to what is seen with CD112 [97]. It was found that CD226 was downregulated in TIGIT^+^CD8^+^ follicular lymphoma T cells compared with TIGIT^−^CD8^+^ cells [50], which further promoted the immune escape process of tumor cells. As a result, Jin, Z., et al. found that patients with acute myeloid leukemia (AML) tended to have fewer CD226^+^ and more TIGIT^+^ γδ T cells [106]. Furthermore, the subset of TIGIT^+^/CD226^−^ γδ T cells was associated with a better prognosis for AML patients, suggesting TIGIT/CD225 might be a promising therapeutic target [106]. In addition, CD226 expression is associated with clinical outcomes in patients with NSCLC treated with an anti-PD-L1 antibody, and PD-1 reduces phosphorylation of both CD226 and CD28 via its ITIM-containing intracellular domain (ICD) [107]. Without CD226 expression, CD8^+^ TILs were unable to respond to anti-PD-1, and thus immune checkpoint blockade efficacy was revealed to be ineffective in CD226^−^ mice [103, 107].

The efficacy of immunotherapy is influenced by the equilibrium between CD226 and TIGIT, given their competing interactions with CD155. Using combined immunotherapy that targets PD-1 and the glucocorticoid-induced tumor necrosis factor receptor-related protein (GITR) antibodies, Wang and colleagues exhibited a survival advantage by ameliorating this balance [108]. Mechanistically, CD226 was reactivated by PD-1 inhibition, whereas TIGIT expression was reduced by GITR [108]. The number of infiltrating suppressive Tregs and the clinical effectiveness of immune checkpoint blockade treatment were both negatively correlated with a greater TIGIT/CD226 ratio in Tregs, which indicates higher TIGIT expression and lower CD226 expression [109].

The majority of T cells, NK cells, and NK/T cells express CD96, an Ig-superfamily receptor, and it can serve as a cancer stem cell marker in AML [110–112]. The CD8^+^ T cells co-express CD96 and PD-1 [113, 114]. However, its function remains unclear. CD96 was demonstrated to enhance the activation of NK cells by increasing adhesion to PVR-expressing target cells [115]. Conversely, NK cell function may be directly inhibited by CD96^+^ NK cells, which is characterized by functional exhaustion and predicts poorer clinical outcomes [116, 117].

Specifically, it was discovered that the novel ligand Nectin-4 (PRR4, PVRL4) only interacts with TIGIT, leading to the inhibition of NK cells [118]. Several cancer cells express Nectin-4 exclusively, as opposed to other nectins [119]. The specific antibody against Nectin-4 enhanced its killing effect on tumor cells in vitro and in vivo [118].

The role of microorganisms in the TME has come under more and more scrutiny in recent years. Fibroblast activation protein 2 (Fap2), which directly interacts with TIGIT in human NK and T cells to cause immunosuppression, has been discovered to be secreted by *Fusobacterium nucleatum*, a bacterium linked to colorectal cancer (Fig. 1) [120]. This discovery has unveiled a novel target for the therapy of microorganisms.

## TIGIT in preclinical studies

Previous studies suggested that reduced tumor burden and survival benefits were found in TIGIT-deficient mice [60, 121]. As such, TIGIT has been identified as a potential star immune checkpoint molecule that inhibits tumor growth and even helps to reduce resistance to immune checkpoint inhibitors in mouse models [25]. In this section, we primarily consolidate the findings from preclinical studies that assess the potential of anti-TIGIT therapy in treating both solid and hematological malignancies. Table 1 presents some of the main preclinical studies on TIGIT.

**Table 1:** Selected Preclinical studies of anti-TIGIT agents in solid and hematological tumors

Tumor types	Main treatment	Dose/Plan (Main treatment)	Object	Description	Other treatment included	Conditions
Solid tumors	Anti-TIGIT (13G6) [122]	250 μg/ twice weekly for 12 weeks (40w-52w)	HBs-HepR mice^a^	Treatment with anti-TIGIT mAb delayed tumor growth, improved the overall survival, maintained a significant response and reinvigorated intrahepatic CD8^+^T cells with increased TNF-α and IFN-γ production and an increased number of CD8^+^T cells.	anti-PD-L1 (10F.9G2)	Hepatocellular Carcinoma
	TIGIT mAb (BE0274, Bio X Cell) [123]	10 mg/kg i.p. three times a week from day 12 to day 40	Tumor bearing *Tgfbr1/Pten* 2cKO mouse models after 5 consecutive days of tamoxifen administered	Treatment with anti-TIGIT slowed down the tumor progression with tolerable cytotoxicity, decreased the infiltration of Tregs, increased frequency of tumor-infiltrating CD8^+^ T cells and CD4^+^ T cells, and abrogated the immunosuppressive capacity of MDSCs relies on CD8^+^ T cells and Tregs.	PD-1 mAb (BE0146; Bio X Cell)	Head and Neck Squamous Cell Carcinoma
	Ociperlimab (BGB-A1217 or BGB-A1217MF) Anti-mouse TIGIT mAb (mu10A7) [124]	10 mg/kg (single agent) or 3 mg/kg (combination with anti-PD-1) i.p. every five days (Q5D) or 5 mg/kg i.p. every one week (QW)	CT26, MC38 or Renca bearing human TIGIT knock-in or BALB/c mouse model	Treatment with BGB-A1217 enhanced IFN-γ secretion of T cells, decreased the infiltration of Tregs, activated NK cells and monocytes and augmented T cell response and showed antineoplastic efficacy in combination with anti-PD-1.	Anti-mouse PD-1 antibody (Ch15mt)	Colon Cancer Kidney Cancer
	COM902 [91]	10 mg/kg (COM902 and anti-mPVRIG) and 3 mg/kg (anti-PD-L1) i.p. on day 6 (monotherapy) or day 8 (combination therapy) post tumor inoculation and dosed three times a week for two weeks	CT26 or Renca cells bearing female Balb/c mouse model	Chimeric COM902 combined with anti-PVRIG or anti-PD-L1 inhibited tumor growth and showed survival benefit.	Anti-PVRIG mAb (COM701) Anti-PD-L1 (Pembrolizuma) Anti-mouse PVRIG (mPVRIG)	Colon Cancer Renal Cell Carcinoma
	Anti-TIGIT [125]	Anti-TIGIT (25 mg/kg), anti-PD-L1 (10 mg/kg), i.p. three times per week for 3 weeks	CT26 and EMT6 cells bearing BALB/c mouse model	Treatment of the combination of anti-TIGIT and anti-PD-L1 enhanced CD8^+^ T cell effector function which could be abrogated by the antibody of CD226.	Anti-PD-L1 Anti-CD226	Colon Cancer Breast Cancer
	Anti-TIGIT (clone 4B1 mIgG2) [126]	200 μg i.p. per animal every other day for 5 doses (anti-TIGIT) or 3 doses (antI-PD-1)	GL261 cells bearing C57BL/6 J mouse model	Treatment with anti-PD-1 and anti-TIGIT improved survival, enhanced effector T cell function and reduced the infiltration of DCs and Tregs.	Anti-PD-1(4 H2)	Glioblastoma
	Anti-TIGIT (Absolute Antibody; Clone 1B4; Mouse IgG1) [127]	100 μg (anti-TIGIT) or 200 μg (anti-PD-1)/mouse i.p. every 2–3 days 100 μg (CD40 agonist) /mouse i.p. once every 4 weeks	Tumor bearing C57BL/6 J mouse model	Treatment of TIGIT/PD-1 co-blockade plus CD40 agonism showed significant anti-tumor function.	anti-PD-1 (BioXCell; Clone 29F.1A12; Rat IgG2a) CD40 agonist (BioXCell; Clone FGK4.5/FGK45; Rat IgG2a)	Pancreatic Cancer
	Anti-TIGIT (#71340, 5 μg/mL, BPS Biosciences) [68]	Not mentioned	CD8^+^ T cells from human gastric cancer tissues	Treatment of anti-TIGIT or combination of anti-TIGIT and SOX enhanced the proliferation and IFN-γ production ability of CD8+ T cells.	S-1 plus oxaliplatin (SOX)	Gastric Cancer
	Anti-TIGIT therapy (clone IG9; BioX Cell, West Lebanon, NH, USA) [128]	Anti-TIGIT: 200 μg i.p. every 3 days for 4 injections Radiotherapy: 15Gy (1.65 Gy/min)	MC38, LLC, B16-F10 tumor cells bearing C57BL/6 mouse models	Administration of the anti-TIGIT enhanced the efficacy of radiotherapy by a CD8^+^ T cell-dependent manner. The combination treatment increased the infiltration of DCs. In addition CD103^+^ DCs were proved to promote the anti-tumor effects of combination therapy.	Radiotherapy	Colon Cancer Lung Cancer Melanoma
	TIGIT-Fc-LIGHT (Bio X Cell, AdipoGen Life Sciences, and LSBio) [129]	200 μg (TIGIT-Fc-LIGHT) or 100 μg (others) i.p. every 3 days for 3 times (mouse model), 0.1 mg/kg, 1 mg/kg, 10 mg/kg or 40 mg/kg i.v. every 7 days for 4 doses (cynomolgus macaques)	CT26/WT, CT26/AR, and B16-F10 tumor bearing BALB/c or C57BL/6 mouse model cynomolgus macaques	TIGIT-Fc-LIGHT exerted anti-tumor efficacy in mouse models regardless of the resistance to PD-1 blockade and showed tolerable toxicity in cynomolgus macaques.	Anti-PD-1 (clone RMP1–14), anti-PD-L1 (clone 10F.9G2), anti-TIGIT (clone 1G9), anti-LTbR (clone 4H8 WH2), TIGIT-Fc, Fc-LIGHT	Colorectal Cancer Melanoma
	Anti-mouse TIGIT (4B1) [34]	200 μg (anti-TIGIT) or 0.5 μg (IL15)/ 3.0 μg (IL15Ra) i.p. on day 0 and day 3, 250 mg (anti-CD226) on days − 1, 0 and 7	B16-F10 or LWT1 tumor bearing C57BL/6 WT or *Tigit*^−/−^ mouse model	The combination of IL15/IL15Ra and anti-TIGIT slowed down the tumor metastasis.	IL15/IL15Ra Complexes (R&D Systems) Anti-CD226 mAb (480.1)	Melanoma
	VV- scFv- TIGIT^b^ [130]	200 μg (anti-PD-1) i.p. every 2 days for 6–7 doses 200 μg (anti-LAG-3) i.p. every 4 days for 3 doses	CT26, MC38,4 T1, H22 bearing BALB/c or C57BL/6 mouse model	The intratumoral injection of VV-scFv-TIGIT elicited anti-tumor function, prolonged survival, increased T cells infiltration and activation of CD8^+^ T cells. Combination of anti-PD-1 or anti-LAG-3 enhanced the anti-tumor efficacy.	Anti-PD-1 (αPD1, Clone RMP1–14, Cat# BE0146, BioXCell) Anti-LAG3 (αLAG- 3, Clone C9B7W, Cat# BE0174, BioXCell)	Colorectal Carcinoma Breast Cancer Hepatocellular Carcinoma
Hematological tumors	Anti-TIGIT (4B1, Bristol-Myers Squibb) [60]	200 μg i.p. / twice per week for 4 weeks from week 4	Vk12653, Vk12598 or 5TGM1 bearing C57BL/6 and TIGIT^−/−^ mouse models	The application of an anti-TIGIT mAb decreased tumor burden, extended survival in the mouse models, and markedly amplified cytokine production along with degranulation of CD8^+^ T cells from MM patients.	Anti-PD-1 (RMP1–14; Bio X Cell)	MM
	Anti-TIGIT (4B1, Bristol Myers Squibb) [61]	100 μg i.p. / twice per week for 6 weeks	B6.WT mice transplanted with Vk12653	Anti-TIGIT prolonged myeloma control after SCT	Not available	Myeloma
	Anti-TIGIT (A15153G, BioLegend) [131]	Cells incubated with 50 μg/mL anti-TIGIT antibody in 96-well plates	TF-1, OCI-ALM3 and MV-4-11 cells	Single or combined with anti-CD39 or A2AR augmented the function of NK-92 cells or the lysis of AML cells	Anti-CD39 Anti-A2AR	AML
	Anti-TIGIT (A15153G; BioLegend) [132]	Cells incubated with 50 μg/mL anti-TIGIT antibody in 96-well plates	AML associated TIGIT^+^ M2 or M1 macrophages	Anti-TIGIT mediated the phenotypic polarization transition from M2 to M1 and increased secretion of cytokines and chemokines associated with the M1 type and enhanced the phagocytosis induced by anti-CD47	Anti-CD47 (CC2C6, BioLegend)	AML

^a^Immunocompetent Fah^−/−^ mouse models of spontaneous HBV-HCC generated by replacing wild-type hepatocytes with HBsAg^+^ hepatocytes

^b^A recombinant engineered oncolytic vaccinia virus (VV) encoding a single-chain fragment (scFv) against TIGIT

*Abbreviation*: *AML* Acute Myeloid Leukemia, *DCs* Dendritic cells, *IFN-γ* Interferon-γ, *mAb* Monoclonal antibody, *MDSCs* Myeloid-derived Suppressor Cells, *MM* Multiple Myeloma, *NK* Natural Killer, *Treg* Regulatory T cell, *TNF* Tumor Necrosis Factor, *scFv* Single-chain Fragment variable, *SCT* stem cell transplantation, *SOX* S-1 plus oxaliplatin

### Solid tumors

**Fig. 4:**
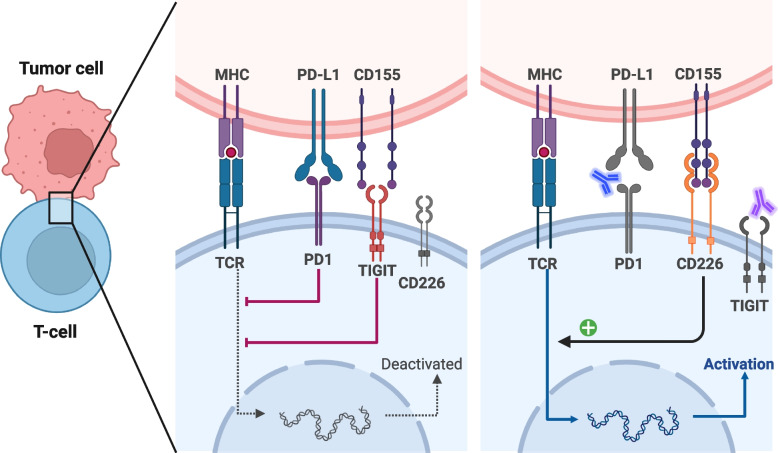
Functions of antibodies targeting TIGIT /and PD-1/PD-L1. The left figure shows the natural immunosuppression and the general ‘lock and key’ structure of the binding of TIGIT and CD155. Besides, the interaction between PD-1 and PD-L1 contributes to immunosuppression as well. Thus, the combination of anti-TIGIT and anti-PD-1/PD-L1 or bispecific antibodies targeting TIGIT and PD-1/PD-L1 is promising, as shown in the right figure. This figure is adapted from “T-cell Deactivation vs. Activation”, by BioRender.com (2020). Retrieved from https://app.biorender.com/biorender-templates

The crux of immunotherapy mainly hinges on the activation of T cells, a pivotal process in marshaling the immune response against malignancies. Accordingly, treatment with anti-TIGIT exerts anti-tumor function by activating CD8^+^ T cells, enhancing the cytotoxicity effects and population of T cells against cancer [66, 122, 123, 133] (Fig. 4). The results from He et al. identified TIGIT blockade to neutralize inhibited T-cell metabolism and IFN-γ production caused by CD155 in gastric cancer cells [26]. Moreover, TIGIT blockade augmented CD8^+^ T-cell reactions and showed a survival benefit in vivo, which was better when targeting TIGIT and PD-1 [26]. The mechanisms could be that TIGIT blockade reversed the suppressed glucose metabolic activity of T cells, induced apoptosis, and reduced G2/M transit in tumor cells [57]. Interestingly, the blockade of TIGIT specifically affects CD8^+^ T cells that express high levels of CD226 by enhancing the phosphorylation at tyrosine 322 of CD226 [104], and increasing the population of CD226^hi^CD8^+^ T cells improves the effects of anti-TIGIT or anti-PD-1 [104].

Tregs play a major role in the occurrence and development of tumors by restricting adaptive immunity; thus, anti-TIGIT therapies targeting Tregs show great promise [31]. It is reported that anti-TIGIT treatment exerts anti-tumor function by decreasing or depleting FoxP3^+^ Tregs [28, 123]. Intriguingly, another study revealed that the TIGIT-blocking antibodies with functional Fc binding did not perform function by depleting Tregs but were mediated by “reverse activating signals” through Fcγ receptors (FcγRs) on myeloid cells, which caused the expression of cytokines and chemokines in colorectal cancer [134].

A unique feature of the anti-TIGIT approach is that it functions not only on the effects of the anti-tumor responses of T cells but also of NK cells. NK cell-mediated immunotherapy is under active development [135–139]. Blocking TIGIT may represent a new approach to enhancing NK function [140]. TIGIT inhibition increased CD56^dim^ NK cell activation in response to the OC tumor [105]. Another study showed that anti-TIGIT prevented NK cell exhaustion and improved its immunogenicity in mouse models, resulting in tumor-specific T cell immunity and magnifying effects of combination immunotherapy with PD-1 and PD-L1 [141]. It is surprising that blocking TIGIT maximized the trastuzumab-induced antitumor response by NK cells in breast cancer [32].

Recently, a pre-clinical study explored the mechanisms of BGB-A1217 (Ociperlimab), a new anti-TIGIT monoclonal antibody (mAb) [124]. This study demonstrated that BGB-A1217 competitively binds to TIGIT with high affinity (K(D) = 0.135 nM), resulting in blocking the binding of TIGIT and its ligands, CD155 or CD112 [124]. In addition, BGB-A1217 induced antibody-dependent cellular cytotoxicity (ADCC) against Tregs, boosted the functionality of NK cells and monocytes, and removed TIGIT from T cell surfaces in an Fc-dependent manner [124]. Although TIGIT mAbs have demonstrated potency in pre-clinical models, the dual antibody combination of anti-TIGIT and anti-PD-1 has garnered more attention due to its superior efficacy.

TIGIT and PD-1 are coinhibitory receptors frequently co-expressed on TILs with exhaustion function [51]. The findings that blocking PD-1/PD-L1 signaling increases the expression of TIGIT on TILs and alters the phenotype of TIGIT^+^ T subsets provide a theoretical basis for the co-blockade strategy [9, 123, 142]. Consistent with this, it was found that blocking TIGIT and PD-1 could increase cytokine production and improve the proliferation of CD4^+^ and CD8^+^ T cells in tumors [91, 143, 144]. In vitro studies showed that this double blockade improved the functionality of CD8^+^ TILs that have no responsiveness to single PD-1 blockades [144]. Besides, the co-blockade also stimulated the CD226 pathway, thereby promoting the T-cell response against tumors [107]. Accordingly, this synergistic treatment could be mitigated by blocking CD226, whose dimerization is disrupted upon direct interaction with TIGIT in a *cis* configuration [125].

Above all, the most standard measure for evaluating the effectiveness of a treatment is the acquired survival benefit. Hung et al. identified that dual therapy resulted in improved survival rates when compared with control or monotherapy, possibly by activating T cell function and downregulating Tregs in mouse models [126]. It significantly reduced tumor progression, ameliorated the poor response of anti-PD-1 therapy, increased the ratio of cytotoxic to regulatory T cells in tumors, and prolonged survival [142, 145]. CD40, expressed on various antigen-presenting cells (APCs), plays a vital role in preserving T cell functionality, and agonistic CD40 antibodies circumvent the need for assistance from CD4^+^ T cells, which provides a novel strategy for targeting poor-responsive tumors [127, 146]. Herein, William et al. administered animal models with triple treatment and received significant tumor responses (46% objective response rate (ORR), 71% disease control rate (DCR), and 23% complete responses (CR) [127]. Importantly, the inclusion of TIGIT blockade could potentially overcome any pre-existing or developed resistance to anti-CD40/PD-1 therapy [127]. Another interesting study found that pH-modulating injectable gel (pH(e)-MIG) increased infiltrating CD8^+^ T cells and thus restored immunosuppression in combination with anti-PD-1 and anti-TIGIT [147]. In addition, the expression of CD96 could be an alternative biomarker for dual blockade efficacy [143]. The introduction of anti-CD96 to dual blockade therapy involving anti-PD-1 and anti-TIGIT resulted in markedly improved responses [113].

Several studies explored the combination of chemotherapy and radiotherapy with anti-TIGIT. On CD8^+^ T cells, the expression of TIGIT was reduced after chemotherapy [68], whereas radiotherapy increased the expression of TIGIT [71, 128]. It is reported that CD103^+^ DCs may play a role in that combination therapy [128]. On the contrary, the combination of anti-TIGIT and chemotherapy enhanced CD8^+^ TIL proliferation and production [68].

A matrix metalloproteinase 2 (MMP-2)-degradable hydrogel that contains doxorubicin (DOX) and blocks of TIGIT has been found to elicit an immunogenic TME and reverse the exhaustion of NK and effector T cells. This led to not only durable localized tumor inhibition but also systemic and long-lasting immune memory responses [148]. As mentioned above, radiotherapy alters the expression of TIGIT and exerts a stronger function in combination with anti-TIGIT or the dual blockade (anti-TIGIT and anti-PD-1). In turn, the use of the anti-TIGIT antibody boosted the efficacy of radiotherapy as well [128]. It was also observed that the dual therapy synergistically extended the accumulation of tumor-infiltrating DCs, which activated CD8^+^ T cells and altered the morphology of myeloid cells in the TME [71, 128, 149]. Building upon this, the incorporation of a triple therapy regimen comprising radiotherapy, anti-PD-1, and anti-TIGIT has presented promising results in various tumor-bearing models, despite the fact that optimizing the radiotherapy strategy required careful consideration to ensure its effectiveness. It was observed that low-dose radiation reduced the expression of CD155 in TAMs and DCs, leading to a decrease in the percentages of TIGIT^+^ exhausted T-cells and TIGIT^+^ Tregs [150, 151].

The TIGIT-Fc fusion protein has received wide attention these years, but the exact effects on tumor immunity remain unclear [129, 152, 153]. It was found that TIGIT-Fc treatment amplified the effects of CD8^+^ T and NK cells in xenograft mouse models [152]. When combined with anti-PD-L1, it promoted the reactivity and sustained memory of tumor immunity via the development of Th1 in CD4^+^ T cells [152].

TNFSF14 is a member of the TNF ligand family, which activates lymphoid cells and triggers the apoptosis of various tumor cells. Recently, an engineered fusion protein, TIGIT-Fc-LIGHT, the linkage between the extracellular domain of TIGIT and the extracellular domain of TNFSF14, was reported to show anti-tumor activity in pre-clinical models, especially in those who were resistant to anti-PD-1 treatment [129].

IL-15 attracted attention in three studies [33, 34, 154]. It was found that IL-15 increased the expression of TIGIT and CD226 on TiNKs, augmented NK-cell-mediated melanoma cytotoxicity in vitro, and suppressed tumor metastasis in preclinical models together with TIGIT blockade [34, 154]. Dogs with metastatic osteosarcoma receiving inhaled IL-15 also exhibited upregulation of activation markers and TIGIT [33]. But more in-depth research is needed.

Furthermore, anti-TIGIT in combination with CD112R combinations [155], CD226 agonists [104], anti-hipoxia-inducible factor 1 (HIF-1)α [156], focal adhesion kinase (FAK) inhibitors [157], and DNA methyltransferases (DNMT) inhibitors [158] are potential therapy strategies. Remarkably, the classic nonsteroidal anti-inflammatory drug aspirin was found to elicit an anti-tumor effect by reducing the expression of TIGIT [159].

Li P. et al. demonstrated that vitamin D was correlated with the expression of TIGIT [160]. Subsequent experiments showed that 1α,25(OH)(2)D(3) inhibited the expression of TIGIT by promoting the binding of vitamin D receptors and the promotor region of TIGIT [160]. In addition, CD8^+^ T cells treated with 1α,25(OH)(2)D(3) increased the functions of production and anti-tumor immunity [160]. Above all, the cytokine production of CD8^+^ T cells could be intensified by oral 1α,25(OH)(2)D(3) [160], which may serve as a potential therapy.

Since oncolytic virus (OV), especially herpes simplex virus (HSV), adenovirus (ADV), and vaccinia virus (VV), the three engineered viruses, have been evaluated for their effect on cancer treatment in pre-clinical and clinical trials and showed a positive response [161, 162], OV has become a new immunotherapy method for some reasons [130] including the ability to increase tumor-specific effector and memory T cells currently [130, 162, 163]. It is reported that the combination of OV and ICIs was better than monotherapy in melanoma [164] due to the immunosuppressive characteristics of TME [165, 166]. On the strength of the former theoretical result, an engineered HSV that expressed a single-chain fragment variable (scFv) against PD-1 enhanced the anti-tumor immunity [167], Zuo, S. et al. designed a VV carried with scFv targeting TIGIT [130]. This engineered VV worked as a virus, replicated in tumor cells, lysed tumor cells, and secreted the anti-TIGIT scFv [130]. Moreover, the VV helped to summon more activated T cells against the immunosuppression in TME [130].

### Hematological tumors

Hematological malignancies form a wide range of tumors that can be categorized as cancers originating in cells of the blood or bone marrow, marked by disturbances and abnormalities in the functioning of the immune system. TIGIT stands out as a highly expressed marker on exhausted NK and T cells, and its high expression was involved in disease progression and the immune escape in various hematological tumors, indicating the promising value of TIGIT blockade treatment [48, 168–173]. To further explore its potential application in the treatment of hematological malignancies, anti-TIGIT has been incorporated into several preclinical studies.

In line with solid tumors, high expression levels of TIGIT characterize the exhausted phenotype of T cells, and blocking TIGIT could decrease FoxP3+ Tregs while stimulating the growth of IFN-γ-producing CD4^+^ T cells and thus confer survival benefits in mouse models [61, 174]. Besides, Mezger et al. concurrently engineered NK-92 cells with knock-out of both CBLB and TIGIT, resulting in enhanced cytotoxicity of NK cells [175]. Also, inhibiting TIGIT/CD155 restored NK cell function in myelodysplastic syndromes (MDS) [176]. As is the case with B cells, TIGIT could curtail B-cell activation and proliferation as a crucial part of an immune shutdown mechanism, which is critical in minimizing immune-induced tissue damage [177]. Contrary to other studies, the research conducted by Tehrani et al. evaluating the combined blocking of TIGIT and PD-1 in CLL did not demonstrate a significant increase in CD8^+^ T cell proliferation and cytotoxicity compared to single treatments [178].. Considering the infiltration of TIGIT^+^ cells was associated with the response to TIGIT blockade [174], the heterogeneity of TIGIT expression should be given careful consideration in future research endeavors [168].

Given its pathogenesis, leukemia typically demonstrates a high degree of motility and the ability to metastasize easily, bypassing the substantial mutational burden frequently encountered in solid tumors [179]. To date, numerous studies have indicated that elevated TIGIT expression is associated with a higher risk of relapse and poor outcomes in AML, regardless of whether allogeneic hematopoietic stem cell transplantation (allo-HSCT) has been performed [180–182]. In addition, blocking TIGIT could serve as a sentisizer for increasing the anti-leukemic effects in AML [95].

Leukemia-associated macrophages (LAMs) constitute a significant cell population within the tumor microenvironment. Using bone marrow (BM) and blood aspirates from AML patients, Fiedler et al. found that TIGIT^+^ M2 LAMs seemed to contribute to an intermediate or adverse risk [132]. Remarkably, in vitro studies have demonstrated that the utilization of anti-TIGIT possesses the potential to mediate the phenotypic polarization transition from M2 to M1, a phenomenon scarcely reported within the context of solid tumors [132]. This also led to increased secretion of cytokines and chemokines associated with the M1 type and enhanced the phagocytosis induced by anti-CD47 [132]. Comparing BM and peripheral blood cells (PBCs) between AML patients and healthy donors (HDs), Fiedler and colleagues discovered that TIGIT was specifically expressed on CD56^dim^CD16^+^ NK cells from AML [131]. They further demonstrated that blocking TIGIT alone could enhance the killing of AML cells mediated by NK-92, with the effect being amplified when combined with anti-CD39 or A2AR inhibitors [131].

## Clinical trials of anti-TIGIT agents

The anti-TIGIT agents registered in clinical trials are listed in Table 2. Phase III clinical trials have been conducted to assess four anti-TIGIT agents: tiragolumab, vibostolimab, domvanalimab, and ociperlimab. Up to this point, the results of three clinical trials evaluating this kind of agent have been published [18–20].

**Table 2:** Ongoing clinical trials of anti-TIGIT agents

Cancer types	Name (manufacturer)	Description	Phases	Conditions	NCT Number
Solid tumors	Domvanalimab (Arcus Biosciences)	IgG1 monoclonal antibody	Early Phase 1	Glioblastoma	NCT04656535
			Phase 2	NSCLC	NCT04262856
					NCT04791839
					NCT05676931
				Upper Gastrointestinal Tract Malignancies	NCT05329766
				Melanoma	NCT05130177
			Phase 3	NSCLC	NCT04736173
					NCT05502237
					NCT05211895
				Advanced Upper Gastrointestinal Tract Malignancies	NCT05568095
	Ociperlimab (BeiGene)	IgG1 monoclonal antibody	Phase 1	Solid Tumors	NCT04047862
			Phase 2	Biliary Tract Carcinoma	NCT05023109
				ESCC	NCT04732494
				NSCLC	NCT05014815
				TNBC	NCT05809895
			Phase 3	NSCLC	NCT04746924
					NCT04866017
	Vibostolimab (Merck)	IgG1 monoclonal antibody	Phase 1	Advanced Solid Tumors	NCT02964013
			Phase 1/2	Melanoma	NCT04305041
					NCT04303169
					NCT04305054
				Prostate Cancer	NCT02861573
				Renal Cell Carcinoma	NCT04626479
			Phase 2	Solid Tumors	NCT05007106
				NSCLC	NCT04165070
				Colorectal Cancer	NCT04895722
			Phase 3	Lung Cancer	NCT04738487
					NCT05298423
					NCT05226598
				SCLC	NCT05224141
				Melanoma	NCT05665595
	Tiragolumab (Roche)	IgG1 monoclonal antibody	Phase 1	Colorectal Cancer	NCT04929223
				Urothelial Carcinoma	NCT05394337
			Phase 1/2	SMARCB1 or SMARCA4 deficient tumors	NCT05286801
				Melanoma	NCT05116202
				SCCHN	NCT05459129
				Urothelial Cancer	NCT03869190
				GA or GEJ or EC	NCT03281369
				Liver Cancer	NCT04524871
				PDAC	NCT03193190
				Endometrial Cancer	NCT04486352
				Locally Advanced ESCC	NCT05743504
			Phase 2	SCCHN	NCT03708224
				Rectal Cancer	NCT05009069
				Melanoma	NCT03554083
				NSCLC or Solid tumors	NCT03977467
				Non-Squamous NSCLC	NCT04958811
				NSCLC	NCT04832854
				Solid Tumors	NCT05483400
				Renal Cell Carcinoma	NCT05805501
			Phase 2	Solid Tumors	NCT05661578
				Squamous Cell Carcinoma of the Anal Canal	NCT05661188
			Phase 2/3	Non-Squamous NSCLC	NCT04619797
			Phase 3	NSCLC	NCT04294810
					NCT04513925
				ESCC	NCT04543617
	Etigilimab (Mereo BioPharma)	IgG1 monoclonal antibody	Phase 1b/2	Solid Tumors	NCT04761198
			Phase 2	Ovarian Cancer	NCT05715216
	EOS-448 (iTeos Belgium SA)	IgG1 monoclonal antibody	Phase 1	Solid Tumors	NCT04446351
			Phase 1/2	Solid Tumors	NCT05060432
			Phase 2	NSCLC	NCT03739710
	HLX301 (Henlius Biotech)	IgG1 Anti-PDL1 and Anti-TIGIT Bispecific Antibody	Phase 1/2	Solid Tumors	NCT05102214
	M6223 (Merck)	IgG1 monoclonal antibody	Phase 1	Solid Tumors	NCT04457778
			Phase 2	Urothelial Cancer	NCT05327530
	JS006 or CHS-006 (Junshi Bioscience, Coherus Biosciences)	IgG4 monoclonal antibody	Phase 1/2	Advanced Solid Tumors	NCT05757492
	ASP8374 (Astellas Pharma)	IgG4 monoclonal antibody	Phase 1	Glioblastoma	NCT04826393
	BMS-986207 (Bristol-Myers)	IgG1 monoclonal antibody	Phase 1/2	Solid Tumors	NCT04570839
				Solid Tumors	NCT02913313
	AZD2936 (AstraZeneca)	IgG1 Anti-PD1 and Anti-TIGIT Bispecific Antibody	Phase 1/2	NSCLC	NCT04995523
			Phase 2	Gastric or GEJ Adenocarcinoma	NCT05702229
	HB0036 (Huaota Biopharm)	Anti-PDL1 and Anti-TIGIT Bispecific Antibody	Phase 1/2	Solid Tumors	NCT05417321
	HB0030 (Huaota Biopharm)	IgG1 monoclonal antibody	Phase 1	Advanced Solid Tumor	NCT05706207
	SEA-TGT (Seagen)	IgG1 monoclonal antibody	Phase 1/2	NSCLC	NCT04585815
	BAT-6005 (Bio-Thera)	IgG1 monoclonal antibody	Phase 1	Advanced Solid Tumors	NCT05116709
	PM1021 (Biotheus)	IgG1 monoclonal antibody	Phase 1	Advanced Solid Tumors	NCT05537051
Hematological tumors	Ociperlimab (BeiGene)	IgG1 monoclonal antibody	Phase 1/2	DLBCL	NCT05267054
	Vibostolimab (Merck)	IgG1 monoclonal antibody	Phase 2	Hematological Malignancies	NCT05005442
	Tiragolumab (Roche)	IgG1 monoclonal antibody	Phase 1/2	BCNHL	NCT05315713
	EOS-448 (iTeos Belgium SA)	IgG1 monoclonal antibody	Phase 1/2	MM	NCT05289492
	BMS-986207 (Bristol-Myers)	IgG1 monoclonal antibody	Phase 1/2	MM	NCT04150965
Combination	Tiragolumab (Roche)	IgG1 monoclonal antibody	Phase 1	Advanced/Metastatic Tumors	NCT02794571
	HLX301 (Henlius Biotech)	IgG1 Anti-PDL1 and Anti-TIGIT Bispecific Antibody	Phase 1/2	Solid Tumors or Lymphoma	NCT05390528
	HLX53 (Henlius Biotech)	IgG1 monoclonal antibody	Phase 1	Advanced/Metastatic Solid Tumors or Lymphoma	NCT05394168
	JS006 or CHS-006 (Junshi Bioscience, Coherus Biosciences)	IgG4 monoclonal antibody	Phase 1	Advanced tumors	NCT05061628
	SEA-TGT (Seagen)	IgG1 monoclonal antibody	Phase 1	Advanced Cancer	NCT04254107
	COM-902 (Compugen)	IgG4 monoclonal antibody	Phase 1	Advanced Tumors	NCT04354246
	AB308 (Arcus Biosciences)	IgG1 monoclonal antibody	Phase 1	Advanced Tumors	NCT04772989
	PM1009 (Biotheus)	IgG1 Anti-TIGIT and Anti- PVRIG Bispecific Antibody	Phase 1	Advanced Tumors	NCT05607563
	AK130 (Akeso)	Anti-TIGIT and Anti-TGF-β Bispecific Antibody	Phase 1	Advanced Malignant Tumors	NCT05653284

*Abbreviation*: *NSCLC* Non-small Cell Lung Cancer, *ESCC* Esophageal Squamous Cell Carcinoma, *TNBC* Triple Negative Breast Cancer, *SCLC* Small Cell Lung Cancer, *SCCHN* Squamous Cell Carcinoma of Head and Neck, *GA* Gastric Adenocarcinoma, *GEJ* Gastroesophageal Junction Adenocarcinoma, *EC* Esophageal Cancer, *PDAC* Pancreatic Ductal Adenocarcinoma, *DLBCL* Diffuse Large B Cell Lymphoma, *BCNHL* B-Cell Non-Hodgkin Lymphoma, *MM* Multiple Myeloma

### Clinical trials in solid tumors

A globle phase II clinical trial (CITYSCAPER, NCT03563716) of tiragolumab, a humanized anti-TIGIT monoclonal antibody, in combination with the PD-L1 inhibitor atezolizumab for 135 PD-L1-positive patients suffering from NSCLC, was first reported at the 2020 American Society of Clinical Oncology (ASCO) meeting [183], and published recently [20]. Regardless of the stage, be it primary or in subsequent update analysis, the combination of tiragolumab with atezolizumab consistently demonstrated significantly improved ORR (primary analysis: 31.3% [95% confidence interval (CI) 19.5–43.2] vs. 16.2% [6.7–25.7] *p* = 0.031; update analysis [until Aug 16, 2021]: 38.8% [95% CI 26.4–51.2] vs. 20.6% [10.2–30.9] *p* = 0.013) and progression-free survival (PFS) (primary analysis: 5.4 months [95% CI 4.2–not evaluable] vs. 3.6 months [2.7–4.4], hazard ratio (HR): 0.57 [95% CI 0·37–0.90] *p* = 0.015) over placebo plus atezolizumab [20]. Remarkably, upon conducting a subgroup analysis based on the expression of PD-L1 (using Tumor Cell Proportion Score, TPS), patients exhibiting high PD-L1 expression levels (TPS ≥50%) demonstrated notably superior benefits, further illuminating the potential of this novel treatment [20]. Moreover, the toxicity was tolerable, with no new adverse effects from the combination [20]. This inspired the continuation of a set of phase III studies known as SKYSCRAPER. The results of the SKYSCRAPER-02 trial (NCT04256421), which evaluated tiragolumab in combination with atezolizumab plus carboplatin and etoposide in small cell lung cancer (SCLC), were reported at the 2022 ASCO annual meeting [23]. Unfortunately, there was no statistical difference in PFS or overall survival (OS) on this trial looking at either the primary analysis set, which included 397 patients, or the full analysis set of 490 patients [23]. Furthermore, the SKYSCRAPER-01 study (NCT04294810), which assessed tiragolumab plus atezolizumab (Tecentriq) vs. atezolizumab alone for patients with PD-L1-high locally advanced or metastatic NSCLC, did not meet the co-primary endpoint of PFS, though OS was immature [21, 24]. Additionally, SKYSCRAPER-03 (NCT04513925) and SKYSCRAPER-07 (NCT04543617) [184] trials are ongoing in patients with locally advanced, unresectable stage III NSCLC and unresectable locally advanced esophageal squamous cell carcinoma (ESCC), respectively.

The results of another study, the MORPHEUS-liver study, were recently updated. This is a phase Ib/II randomized evaluation of tiragolumab in combination with atezolizumab and bevacizumab in patients with unresectable, locally advanced, or metastatic hepatocellular carcinoma. According to the latest information from the 2023 ASCO, a total of 58 patients were enrolled. In the tiragolumab + atezolizumab + bevacizumab arm, the ORR was 42.5% vs. 11.1% in the atezolizumab and bevacizumab control arm. Besides, longer PFS was observed in the treatment arm (11.1 months; 95% CI: 8.2–not evaluable vs. 4.2 months; 95% CI: 1.6–7.4) regardless of the expression of PD-L1, and the toxicity was tolerable. However, it was notable that the 11.1% ORR in the control arm appeared significantly lower than that when used as a first-line therapy [185, 186].

The results of a phase I study on vibostolimab, a mouse and human chimeric anti-TIGIT monoclonal antibody, have just been published [18]. The combination of vibostolimab and pembrolizumab showed promising anti-tumor efficacy and manageable toxicity in patients with advanced solid tumors and anti-PD-1/PD-L1-naive NSCLC [18]. Recently, three phase III trials, including KeyVibe-006 (NCT05298423) [187], KeyVibe-007 (NCT05226598) [188] and KeyVibe-008 (NCT05224141) [189], were started to evaluate the efficacy of different strategies, including vibostolimab for NSCLC [187, 188] or SCLC [189], with the results pending.

Contrary to other anti-TIGIT monoclonal antibodies, domvanalimab (D) is an Fc-silent anti-TIGIT experimental monoclonal antibody that does not interact with effector T cells. The primary results of the phase II study, ARC-7, were presented at the 2022 ASCO meeting. Compared with zimberelimab (Z) single-agent treatment (27%; 95% CI: 15.0–42.8), D-containing arms (including DZ and DZ plus etrumadenant, E) acquired improved ORR (DZ: 41%; 95% CI: 26.3–56.8, EDZ: 40%; 95% CI: 25.7–55.7). Of note, D-containing arms observed significant prolonged PFS (DZ: 12 months; 95% CI: 5.5-not evaluable; HR: 0.55 [95% CI: 0.31–1.0], EDZ: 10.9 months; 95% CI: 4.8-not evaluable; HR: 0.65 [95% CI: 0.37–1.1]) in contrast to Z (5.4 months; 95% CI: 1.8–9.6) in patients with high PD-L1 expression (TPS ≥ 50%), EGFR/ALK wild-type, and metastatic NSCLC after median follow-up of 11.8 months [190]. To substantiate these promising findings, the ARC-10 (NCT04736173), a phase III clinical trial evaluating the efficacy of domvanalimab and zimberelimab combination therapy, has been conducted. In addition, the treatment of NSCLC and upper gastrointestinal tract cancer with D monotherapy or in combination with anti-PD-1/PD-L1 is currently being evaluated in four phase III clinical trials, including ARC-10 (NCT04736173), PACIFIC-8 (NCT05211895) [191], STAR-121 (NCT05502237), and STAR-221 (NCT05568095).

A preclinical study on ociperlimab (BGB-A1217), a humanized monoclonal antibody against TIGIT, elicited its competitive inhibition of TIGIT [124]. Then, the safety and efficacy of ociperlimab plus tislelizumab combination therapy were assessed in a phase I study, AdvanTIG-105 (NCT04047862). The findings were reported to be all-dose-tolerable in advanced solid tumors with promising antitumor activity [192]. On top of that, this combination treatment strategy was also conducted in several other clinical studies, including AdvanTIG-202 (phase II) [193], AdvanTIG-203 (NCT04732494, phase II) [194], AdvanTIG-206 (NCT04948697, phase II) [195], and AdvanTIG-302 (NCT04746924, phase III) [196].

### Clinical trials in hematological tumors

In addition to solid tumors, numerous clinical investigations have been focused specifically on hematologic malignancies, as summarized in Table 2. However, none of these studies has yielded definitive results to date. The specific reasons for this lack of conclusive findings remain to be elucidated by researchers in the future.

### Potential factors contributing to clinical failures of anti-TIGIT

Based on the immune checkpoint blockades associated with the mechanism of action of TIGIT [1, 26, 66, 197], we summarized a few possible reasons for the failures of early clinical trials in patients with SCLC and NSCLC.

First, controlling the expression of PD-L1, TIGIT, and other biomarkers, as well as the quantity and quality of TILs, circulating tumor cells, and TAMs, can affect tumor invasion, metastasis, and recurrence in various patient populations [198, 199]. There must be enough immune T cells in the patients to be recruited to tumors for immunotherapy. Tumors with T cell exhaustion, called “cold” tumors, vs. tumors with plenty of T cells, named “hot” tumors, may have different reactions to the TIGIT targeting therapy.

Second, phase II results might not accurately represent the TIGIT-blocking therapy’s true effects. TIGIT, which serves as the “braker” in immune checkpoint regulation, together with another “braker”, PD-1/PD-L1, may not be sufficient to activate the immune cells to kill tumor cells, especially in patients with advanced or high-burden tumors. TIGIT cancer immunotherapy may be made more effective by combining it with additional “braker” drugs, such as anti-CTLA-4, anti-vascular endothelial growth factor (VEGF), a triple combination of TIGIT^+^PD-1/PD-L1^+^CTLA-4 or TIGIT^+^PD-1/PD-L1^+^VEGF, or chemotherapy. Multiple bispecific and trispecific antibodies are currently in clinical development and show promising preliminary therapeutic activity [200].

## Conclusion and future perspectives

Immunotherapy, represented by anti-PD-1/PD-L1, has revolutionized the field of oncology. Since the approval of the first immune checkpoint inhibitor, Ipilimumab, by the FDA in 2014, numerous cancers have been included in clinical trials demonstrating significant responses to this form of treatment. However, the reality is harsh. According to a recent statistical report, approximately only half of the patients treated with PD-1/PD-L1 monotherapy exhibited a response, irrespective of their PD-L1 expression and type of cancer [201]. The challenges of sensitivity and resistance consistently obstruct our progress towards eradicating cancer, much like the hurdles we face with chemotherapy and targeted therapy. As a newly implemented immune checkpoint, TIGIT offers an alternative potential solution to this situation, and the enhanced cytotoxicity of combining treatments, including anti-TIGIT therapies, has been observed in several clinical trials [18, 20].

Indeed, as previously mentioned, the failure of the SKYSCRAPER-02 trial seemed to cast a shadow over this “uncertain” regimen. Meanwhile, unlike anti-PD-1/PD-L1 therapies, an effective biomarker for anti-TIGIT treatment is yet to be identified, which further complicates the situation. Therefore, future research should focus on discovering novel biomarkers or different approaches for targeting TIGIT, such as bispecific antibodies, antibody-drug conjugates, and CAR-T cells targeted at TIGIT.

## Data Availability

All clinical trials related information was obtained from public databases.

## Abbreviations

ADCC: Antibody-dependent cell-mediated cytotoxicity
ADV: Adenovirus
Allo-HSCT: Allogeneic hematopoietic stem cell transplantation
AML: Acute myeloid leukemia
APC: Antigen-presenting cell
ASCO: American Society of Clinical Oncology
BCNHL: B-Cell Non-Hodgkin Lymphoma
BM: Bone marrow
CAR-T: Chimeric antigen receptor T cells
CCL23: C-C motif chemokine ligand 23
CI: Confidence interval
CLL: Chronic lymphocytic leukemia
CR: Complete response
CSR: Chimeric costimulatory switch receptors
CTLA-4: Cytotoxic T lymphocyte antigen-4
DC: Dendritic cell
DCR: Disease control rate
DOX: Doxorubicin
DLBCL: Diffuse Large B Cell Lymphoma
DNMT: DNA methyltransferase
EC: Esophageal Cancer
ESCC: Esophageal squamous cell carcinoma
FAK: Focal adhesion kinase
Fap2: Fibroblast activation protein 2
FcγR: Fcγ receptors
FDA: Food and Drug Administration
GA: Gastric Adenocarcinoma
GEJ: Gastroesophageal Junction Adenocarcinoma
GITR: Glucocorticoid-induced tumor necrosis factor receptor-related protein
HBV-HCC: Hepatitis B virus-associated hepatocellular carcinoma
HD: Healthy donor
HIF-1: Hipoxia-inducible factor 1
HR: Hazard ratio
HSV: Herpes simplex virus
ICI: Immune checkpoint inhibitors
ICD: ITIM-containing intracellular domain
IFN-γ: Interferon-γ
IFN-I: Type 1 interferon
IgV: Immunoglobulin variable-set
ITIM: Immunoreceptor tyrosine-based inhibitory motif
ITT: Immunoglobulin tyrosine tail
LAG-3: Lymphocyte activating gene-3
LAM: Leukemia-associated macrophage
LUSC: Lung squamous cell carcinoma
mAb: Monoclonal antibody
MCL: Mantle cell lymphoma
MDS: Myelodysplastic syndromes
MDSC: Myeloid-derived suppressor cell
MHC: Major histocompatibility complex
MM: Multiple myeloma
MMP-2: Matrix metalloproteinase 2
NHL: Non-Hodgkin lymphoma
NK: Natural killer
NSCLC: Non-small cell lung cancer
OC: Ovarian cancer
ORR: Objective response rate
OS: Overall survival
OV: Pncolytic viruses
PBC: Peripheral blood cell
PDAC: Pancreatic Ductal Adenocarcinoma
PD-1: Programmed cell death protein 1
PD-L1: Programmed death-ligand 1
PFS: Progression free survival
PVR: Poliovirus receptor
SCCAC: Squamous Cell Carcinoma of the Anal Canal
SCCHN: Squamous Cell Carcinoma of Head and Neck
scFv: Single-chain fragment variable
SCLC: Small Cell Lung Cancer
SCT: Stem cell transplantation
SH2: Src homology 2
SHIP1: SH2-containing inositol phosphatase 1
SOX: S-1 plus oxaliplatin
TAM: Tumor-associated macrophage
TFH: Targeting follicular helper T cells
Th: T helper cell
TIGIT: T-cell immunoreceptor with Ig and ITIM domains
TIL: Tumor infiltrating lymphocyte
TIM: Tumor-infiltrating myeloid cell
TIM-3: T cell immunoglobulin and mucin-domain-containing-3
TiNK: Tumor-infiltrating NK cell
TLS: Tertiary lymphoid structure
TME: Tumor microenvironment
TNBC: Triple Negative Breast Cancer
TNF: Tumor necrosis factor
TPS: Tumor Cell Proportion Score
TRAF6: TNF receptor-associated factor 6
Treg: Regulatory T cell
VEGF: Vascular endothelial growth factor
VV: Vaccinia virus
